# Prediction of mechanistic subtypes of Parkinson’s using patient-derived stem cell models

**DOI:** 10.1038/s42256-023-00702-9

**Published:** 2023-08-10

**Authors:** Karishma D’Sa, James R. Evans, Gurvir S. Virdi, Giulia Vecchi, Alexander Adam, Ottavia Bertolli, James Fleming, Hojong Chang, Craig Leighton, Mathew H. Horrocks, Dilan Athauda, Minee L. Choi, Sonia Gandhi

**Affiliations:** 1 Department of Clinical and Movement Neurosciences, UCL Queen Square Institute of Neurology, London, UK; 2 The Francis Crick Institute, King’s Cross, London, UK; 3 Faculty, Marylebone, London, UK; 4 Institute for IT Convergence, KAIST, Daejeon, Republic of Korea; 5 EaStCHEM School of Chemistry, The University of Edinburgh, Edinburgh, UK; 6 IRR Chemistry Hub, Institute for Regeneration and Repair, The University of Edinburgh, Edinburgh, UK; 7 Department of Brain & Cognitive Sciences, KAIST, Daejeon, Republic of Korea

**Keywords:** High-throughput screening, Neurodegeneration

## Abstract

Parkinson’s disease is a common, incurable neurodegenerative disorder that is clinically heterogeneous: it is likely that different cellular mechanisms drive the pathology in different individuals. So far it has not been possible to define the cellular mechanism underlying the neurodegenerative disease in life. We generated a machine learning-based model that can simultaneously predict the presence of disease and its primary mechanistic subtype in human neurons. We used stem cell technology to derive control or patient-derived neurons, and generated different disease subtypes through chemical induction or the presence of mutation. Multidimensional fluorescent labelling of organelles was performed in healthy control neurons and in four different disease subtypes, and both the quantitative single-cell fluorescence features and the images were used to independently train a series of classifiers to build deep neural networks. Quantitative cellular profile-based classifiers achieve an accuracy of 82%, whereas image-based deep neural networks predict control and four distinct disease subtypes with an accuracy of 95%. The machine learning-trained classifiers achieve their accuracy across all subtypes, using the organellar features of the mitochondria with the additional contribution of the lysosomes, confirming the biological importance of these pathways in Parkinson’s. Altogether, we show that machine learning approaches applied to patient-derived cells are highly accurate at predicting disease subtypes, providing proof of concept that this approach may enable mechanistic stratification and precision medicine approaches in the future.

## Abstract (web summary)

Deep learning applied to live-cell images of patient-derived neurons aids predicting underlying mechanisms and gains insights into neurodegenerative diseases, facilitating the understanding of mechanistic heterogeneity. D’Sa and colleagues use patient-derived stem cell models, high-throughput imaging and machine learning algorithms to investigate Parkinson’s disease subtyping.

## Main

Parkinson’s disease (PD) is a progressive neurodegenerative disorder that encompasses several pathogenic processes that converge on the accumulation of misfolded α-synuclein (α-Syn) in Lewy bodies and neurites, and degeneration of dopaminergic neurons in the substantia nigra, resulting in an array of motor, cognitive, neuropsychiatric and autonomic deficits^1–3^. The age of onset, rate of disease progression, and severity of motor and non-motor symptoms display considerable individual variation (see ref. ^4^ for a review). This is most likely due to differences in the underlying molecular mechanisms occurring in different subtypes of the disease (see ref. ^5^ for a review). Critically, there are currently no approaches to define the molecular heterogeneity, and therefore no opportunity to understand the mechanisms that may drive the different phenotypic subtypes. An unmet challenge is to make an early and accurate molecular-level diagnosis of the condition, as this would enable the field to consider targeted interventions appropriate to an individual’s condition, and offer an opportunity to do this at the earliest possible time.

**Fig. 1:**
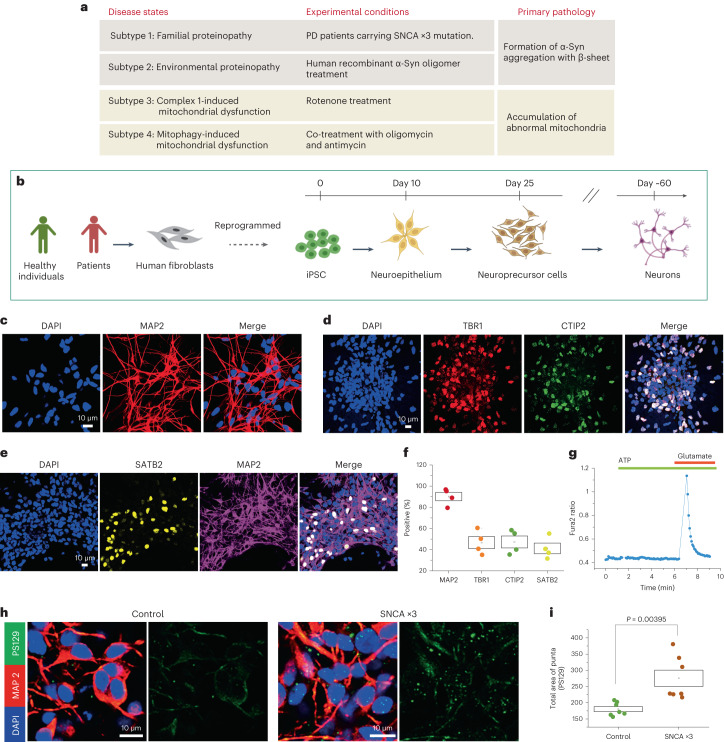
Pathological cellular subtypes of PD and the generation of a human PD model using hiPSCs. **a**, Details on the cellular subtypes. Subtype 1: cells generated with SNCA ×3 mutation represent familial proteinopathy. Subtype 2: environmental proteinopathy was induced by exposing cells to exogenous protein aggregates. Subtype 3: toxin-induced mitochondrial dysfunction was achieved by exposing cells to rotenone, a complex 1 inhibitor. Subtype 4: mitophagy was induced using stimulation with oligomycin/antimycin. **b**, Schematic showing hiPSC-derived neuronal differentiation strategy. Fibroblasts from patients with PD or healthy donors are reprogrammed into hiPSCs and differentiated into cortical neurons using a protocol adapted from ref. ^14^. **c**–**f**, Characterization of iPSC-derived neurons using immunohistochemistry for the representative images of MAP2, a neuronal marker (**c**); TBR1 and CTIP2, deep cortical layers (**d**); SATB2, the upper cortical layer (**e**); and quantification (**f**; *n* = 4 number of wells per group). **g**, Calcium imaging measured with Fura-2 shows that the hiPSC-derived cortical neurons exhibit calcium signals in response to physiological concentrations (5 µM) of glutamate. **h**,**i**, hiPSC-derived neurons from PD patients with SNCA ×3 mutation display an increase in phosphorylated α-Syn (a pathological form of α-Syn) (*n* = 6 or 7 number of wells per group). Statistical details are found in Supplementary Table 4. The data in **i** are presented as data ± s.e.m. Source data

We applied a deep learning approach to human cellular models of PD to generate a predictive model of different mechanisms of disease. Parkinson’s disease is known to be caused by a complex interplay of genetic and environmental drivers. Two common and critical pathways that drive pathology include (1) the accumulation of insoluble aggregates of the protein α-Syn, implying that protein misfolding and impaired protein homeostasis cause a proteinopathy or synucleinopathy^2,6^, and (2) the accumulation of abnormal mitochondria with impaired bioenergetic function and reduced mitochondrial clearance^7^ (see ref. ^8^ for a review). We used patient-derived, induced pluripotent stem cell (iPSC)-derived cortical neurons to model PD: these are a robust preclinical cell model for the disease, recapitulating the human genomic and proteomic environment of a differentiated cell type that is affected in the disease^9–11^. We defined four cellular subtypes that map to both of these two key pathways that lead to disease (Fig. 1a). Subtype 1: patients with mutations in the SNCA gene (gene ID: 6622) encoding α-Syn develop an autosomal dominant aggressive form of Parkinson’s with predominant protein aggregation, which is directly caused by the mutation in the SNCA gene; thus, iPSC-derived neurons from a patient with SNCA triplication (SNCA ×3) were used to model familial proteinopathy. Subtype 2: protein aggregates are known to spread from cell to cell in the brain in a prion-like manner, inducing proteotoxic stress; we exposed healthy iPSC-derived neurons to aggregates of α-Syn to recapitulate the environmental proteinopathy^12,13^. Subtype 3: exposure to pesticides with subsequent mitochondrial complex 1 impairment can induce PD, and patients with PD exhibit widespread impairment of complex 1-dependent respiration; we applied a complex 1 inhibitor, rotenone, to generate a model of toxin-induced mitochondrial dysfunction. Subtype 4: mutations in the PINK1 and PARKIN genes cause autosomal recessive early-onset PD, and these mutations directly result in impaired clearance of damaged mitochondria (mitophagy), resulting in the accumulation of abnormal mitochondria in neurons^8^; we applied another mitochondrial damage stress that is a known inducer of mitophagy—oligomycin/antimycin—to generate another model of mitochondrial dysfunction. After establishing a series of classifiers for disease subtypes that had been genetically and chemically induced, we then tested whether the same approach could be used in a real-world scenario, in which a patient’s genetic status (a carrier of an SNCA or PINK1 mutation) would influence their cellular disease subtype (proteinopathy versus mitochondrial).

We fluorescently labelled specific cellular compartments (the nucleus, mitochondria and lysosomes) while simultaneously performing high-content live single-cell imaging of iPSC-derived neurons. Using data from multiple plates (total 1,560,315 cells), we generated models to predict disease state and disease subtype. We generated two broad types of classifier. First, a prediction classifier based on automatically extracted features (56 features), providing deep profiling of cellular phenotypes: this classifier has the advantage of high explainability using the ranking of features. Second, prediction classifiers based on images and convolutional neural network analyses, which use the power of computer vision to extract large amounts of unbiased information; this classifier has very high accuracy but less explainability.

Our work identifies specific features in neurons that are able to predict different cellular subtypes of the disease, and therefore provides valuable biological insights into the mechanisms of PD.

## Results

### Human iPSC-derived cortical neurons to model disease

Human iPSCs (hiPSCs; see Supplementary Table 1 for details on the iPSC lines) were generated through reprogramming fibroblasts from healthy donors or PD patients carrying SNCA ×3. Neuronal differentiation was performed using a protocol adapted from ref. ^14^ (Fig. 1b). Following terminal differentiation, the culture is highly enriched in neurons, displaying neuronal markers (MAP2 = 90.01 ± 3.516%; Fig. 1c,f) with both lower- (TRB1 = 46.67 ± 4.985%, CTIP2 = 47.30 ± 5.037; Fig. 1d,f) and upper-layer (SATB2 = 41.15 ± 4.501%; Fig. 1e,f) neuronal markers. Neuronal cultures responded to physiological concentrations of glutamate (5 μM), confirming that the majority of the population are glutamatergic neurons (62.92 ± 3.819%; Fig. 1g). A key hallmark of PD is the accumulation of intraneuronal aggregates comprising phosphorylated α-Syn. Human iPSC-derived neurons carrying SNCA ×3 mutation express more phosphorylated forms of α-Syn compared with control iPSC-derived neurons (Fig. 1h,i).

We confirm the generation of human cortical neurons^13,15,16^ and, furthermore, that subtype 1 (familial proteinopathy) exhibits the pathological hallmark of a proteinopathy^13,15,17^.

### Defining and acquiring data on disease states in PD

**Fig. 2:**
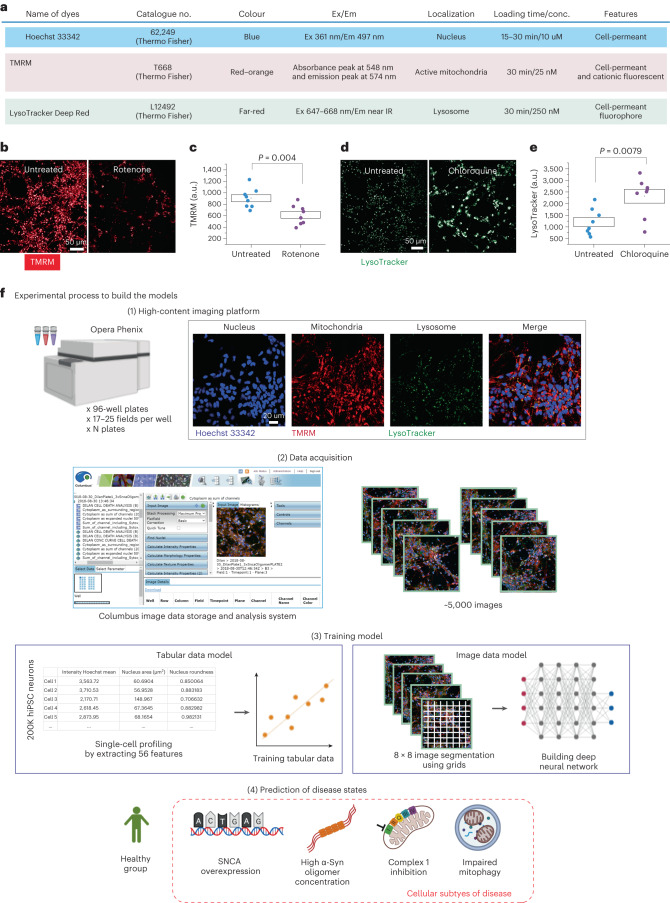
Workflow to develop a classifier to make a prediction of cellular subtypes in PD. **a**, Experimental details for live-cell imaging. **b**–**e**, High-throughput imaging enables visualization of mitochondrial depolarization by complex 1 inhibitor, rotenone (5 µM; **b**,**c**, *n* = 8 number of wells per group) and lysosomal dysfunction by chloroquine (1 µM; **d**,**e**, *n* = 8 number of wells per group). The statistical details are found in Supplementary Table 4. Data in **c** and **e** are presented as data ± s.e.m. **f**, A schematic illustration to describe the experimental process of building the models. First, live-cell imaging with an Opera Phenix High-Content Screening System (PerkinElmer); cells are loaded with live-cell imaging dyes. Representative images for the three channels: Hoechst 33342 (nucleic labelling within 387/11 nm excitation and 417–447 nm emission); TMRM (mitochondrial labelling within 505 nm excitation and 515 nm emission); and LysoTracker deep red (lysosomal labelling within 614 nm excitation and 647 nm emission). Second, a Columbus Image Data Storage and Analysis System (PerkinElmer) was used to extract 56 morphological features (Extended Data Figs. 1a and 2a) and whole images. Third, models are trained on tabular data extracted from cell profiling features or images uniformly gridded by 8 × 8 segmented cropped images and categorically labelled and fed into the neural network. Fourth, the learned model enables the prediction of the healthy group or the four disease subtypes. Source data

**Extended Data Fig. 1:**
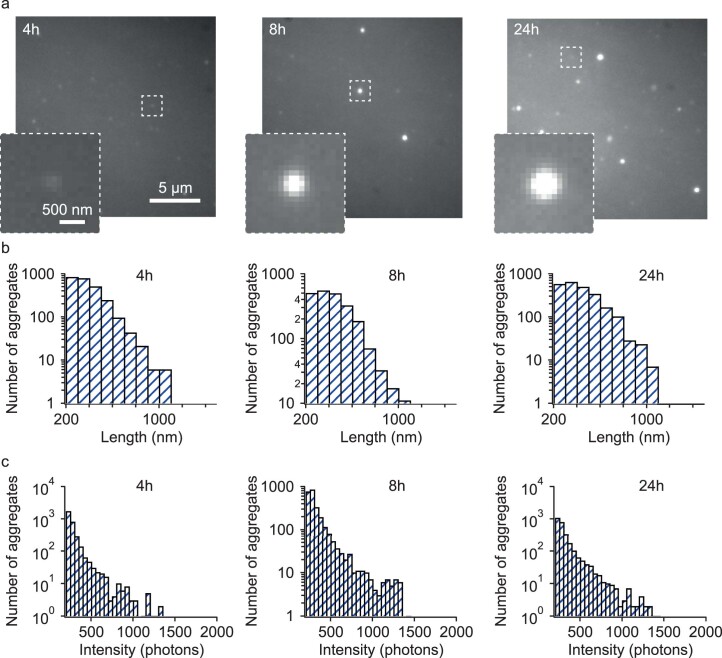
Characterization of α-Syn oligomers using SAVE imaging. (a) Representative SAVE images of early oligomers (4 h), late oligomers (8 h), and fibrils (24 h). The length and intensity of each detected aggregate were determined, and are presented in histograms of lengths (b) and intensities (c). 25 SAVE images were taken for each time point.

Mitochondrial dysfunction and synucleinopathy are two primary pathologies of PD and are induced by various conditions^2,18,19^. We established a set of disease states (four subtypes as described in Fig. 1a) led by two primary pathologies: α-Syn aggregation and mitochondrial dysfunction. Subtype 1 is a familial proteinopathy, generated using neurons from PD patients carrying SNCA ×3, which has elevated aggregation levels in neurons (Fig. 1h,i)^13,15^. Subtype 2 is an environmental proteinopathy, generated by treating control neurons with α-Syn oligomers, a toxic soluble species of ɑ-Syn (see Extended Data Fig. 1a–c for characterization of oligomers)^13,15,17^. Subtype 3 is a mitochondrial dysfunction state induced by inhibiting complex 1 using rotenone (5 µM)^20–22^. Subtype 4 is a mitochondrial dysfunction state that is induced by co-treating with oligomycin A (1 µM) and antimycin (1 µM)^23^. Live-cell imaging of multiplexed dyes (Hoechst; tetramethylrhodamine, methyl ester, perchlorate (TMRM); LysoTracker; and SYTOX green) revealed the organelles of live viable hiPSC-derived neurons (nucleus, mitochondria and lysosomes) in the different disease subtypes. The fluorescent signal of the dyes in live cells is dependent on the physiological status of the organelles (Fig. 2b–e). Rotenone depolarizes mitochondrial membrane potential, resulting in loss of fluorescent intensity of TMRM (*P* = 0.0046; Fig. 2b,c). Chloroquine alters lysosomal activity and induces an increase in the fluorescence of the dye LysoTracker (*P* = 0.0020; Fig. 2d,e).

**Extended Data Fig. 2:**
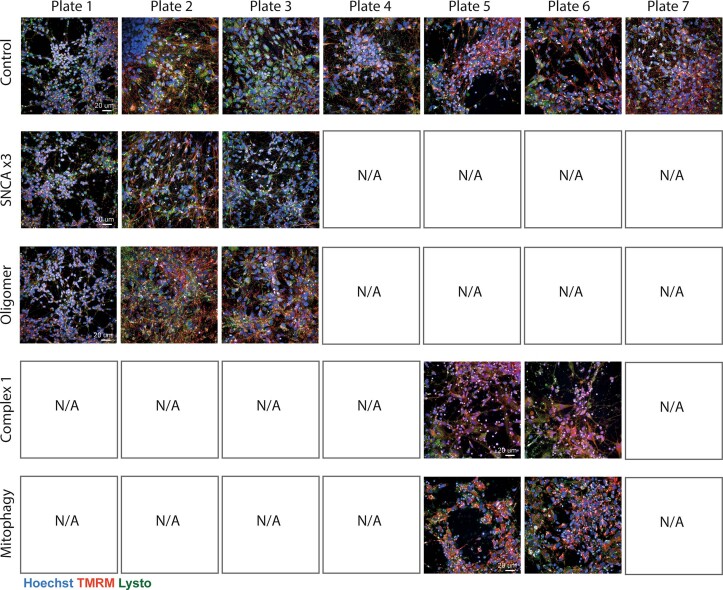
Representative images of the plates used. Representative images show similar image quality across the plates.

**Extended Data Fig. 3:**
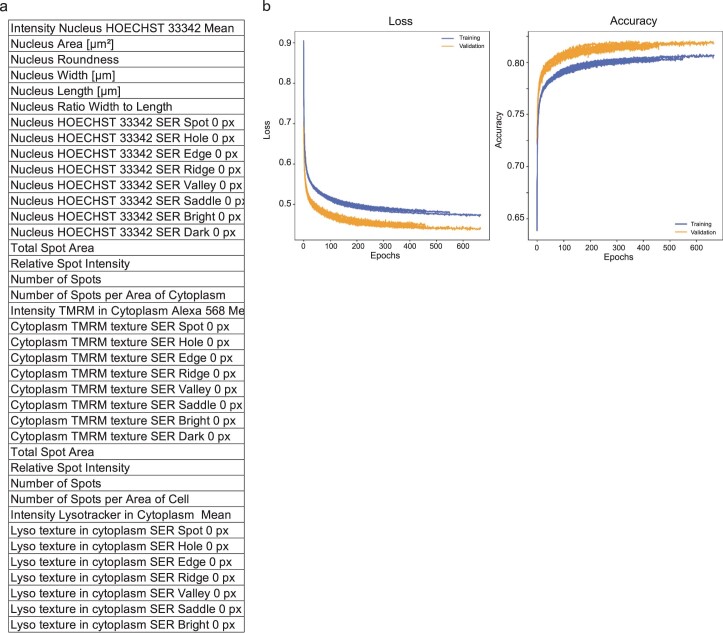
Supportive data for Fig. 3. **a**. The features regarding three key organelles, nucleus (Hoechst3337), mitochondria (TMRM) and lysosome (Lyso) used to train tabular data. Morphologically defined features are included such as cell area, expression intensity, the number of spots, roundness, length and width. SER texture features are also included defined as Spot, Hole, Edge, Ridge, Valley, Saddle, Bright and Dark which measure local patterns of pixel intensity providing the structural information of the organelle loading (reviewed here (Di Cataldo and Ficarra, ^39^) (Cretin et al., ^40^). **b**. The Loss and Accuracy curve (training and validation).

**Extended Data Fig. 4:**
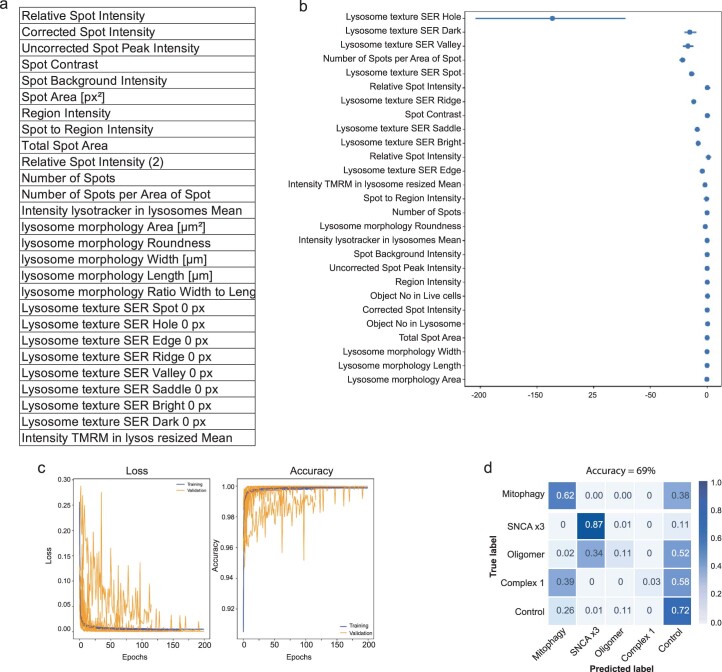
**a**. Cell profiling features for the lysosomal features that contact mitochondria. **b**. The plot shows the average scaling factor, with standard deviation, for control distribution across plates (n = 541,300 cells) for the lysosomal features that contact mitochondria in the training dataset. Data are presented as mean values ± SD. The features were scaled per control in each plate using the Power Transformer scaler. Those with a high variance in the scaling factor, lambda, (>50) in the training dataset, such as ‘Lysosome texture SER Hole’ feature were excluded from the training, validation and test dataset. **c**. ROC-AUG (ci) and the Loss and Accuracy curve (training and validation. **d**. Confusion matrix of 5-class model training on the selected data from the mitochondrial and lysosomal co-localization.

Live-cell images were acquired using an Opera Phenix High-Content Screening System (PerkinElmer; the representative images of each condition are shown in Extended Data Fig. 2) across a range of neuronal inductions (*n* = 8) and different plates (*n* = 11) to capture the inherent variation between cell lines, differentiation of hiPSCs, and the variation in dye loading and cellular imaging (Fig. 2a and Supplementary Tables 2 and 3). All available features were extracted from the Columbus Image Data Storage and Analysis System (Extended Data Figs. 3a and 4a). We acquired the data in two formats: (1) tabular data, which consisted of 56 mitochondrial, lysosomal and nuclear features; and (2) 1,024 × 1,024 raw images, which we further segmented into smaller 8 × 8 tiles (the experimental process to build the models is shown in Fig. 2f).

### A classifier trained on cellular profiles predicts disease

**Fig. 3:**
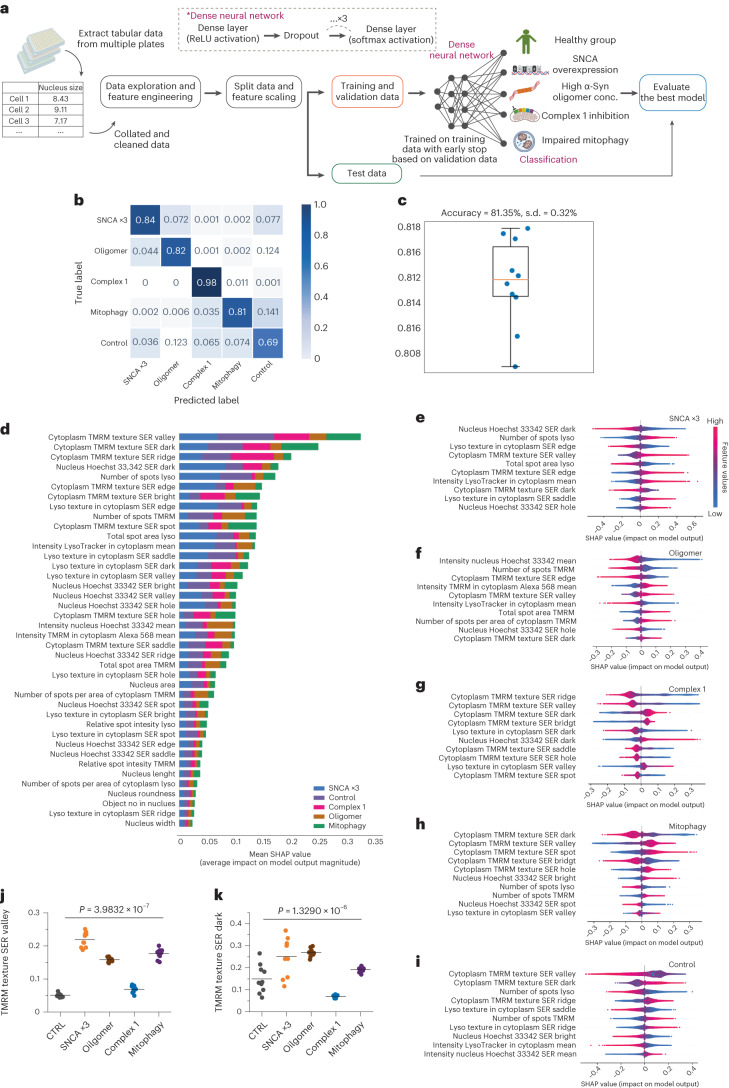
A classifier trained on cell profiles of key organelles predicts disease states with 82% accuracy. **a**, An illustration of workflow for machine learning with tabular data. **b**,**c**, Classification performance by a confusion matrix (**b**) and the stratified K-fold cross-validation (**c**) on an unseen test set, trained on cell profile tabular data (*n* = 10 folds fit and evaluated; data are presented as mean values ± s.d.). **d**, Feature ranking based on their SHAP values coloured by their importance for each class. **e**–**i**, A SHAP summary plot for the top ten most important features based on their SHAP values for each of the classes: SNCA ×3 (**e**), oligomer (**f**), complex 1 (**g**), mitophagy (**h**) and control (**i**). Dots are coloured according to the values of features for each cell; red and blue represent high and low feature values, respectively. A positive SHAP indicates an increased probability of predicting each state (positive impact on the output) and vice versa. **j**,**k**, Random selection of ten wells to test top two features shows an effect of cellular subtype across five groups (one-way ANOVA *P* < 0.0005, *n* = 10 number of wells per group). The statistical details are found in Supplementary Table 4. Data are presented as data and mean. Control, healthy group; SNCA ×3, SNCA mutation; oligomer, treatment with α-Syn oligomer; complex 1, treatment with mitochondrial complex 1 inhibition; mitophagy, co-treatment with antimycin and oligomycin to induce mitophagy. Source data

We built a classifier to predict five classes—four disease subtypes and one healthy state—using tabular data based on the nucleus, mitochondria and lysosome features extracted from the live high-content imaging platform (the experimental workflow is shown in Fig. 3a). We designed, trained and evaluated a dense neural network by splitting the entire dataset (*n* = 1,560,315 identified cells) into training (40%), validation (30%) and test (30%) datasets. The confusion matrix demonstrates that, overall, the correct label was identified 82% of the time in the five-class model. However, although some of the classes had an accuracy close to the model’s overall accuracy (SNCA ×3 = 84%, oligomer = 82%, mitophagy = 81%), specific disease subtypes had very high accuracy, notably complex 1 dysfunction (complex 1 = 98%), whereas the control state had slightly lower accuracy (control = 69%; Fig. 3b and Extended Data Fig. 3b). The accuracy of the model with all five classes was cross-validated using stratified K-fold cross-validation (accuracy 81.35 ± 0.32%; Fig. 3c), where the dataset split was independently randomized ten times. We next explored the feature importance of the model to understand the cellular features that drive accurate prediction using Shapley additive explanation (SHAP) values on the entire test set; SHAP is an estimated value of importance for each feature in the model^24^. The ranked features based on their SHAP values—coloured by their importance for each class—indicate that the majority of the features that explain the model’s predictions originate from mitochondrial terms (TMRM-related features; Fig. 3d; details on the features are described in Extended Data Fig. 3a). This is highlighted for all disease subtypes where TMRM readouts are the highest SHAP-ranked features, followed by the nuclear and lysosomal features (Fig. 3d). We then explored the top ten SHAP features for each disease subtype individually to ascertain which organelle is driving the prediction for each pathway, and to understand the relationship between the feature value and SHAP value (Fig. 3e–i). Importantly, this demonstrates that out of the top ten features for subtype 1 (SNCA ×3), five were lysosomal, three were mitochondrial and two were nuclear (Fig. 3e); for subtype 2 (oligomer), seven were mitochondrial, two were nuclear and one was lysosomal (Fig. 3f); for subtype 3 (complex 1), seven were mitochondrial, two were lysosomal and one was nuclear (Fig. 3g); and for subtype 4 (mitophagy), six were mitochondrial, two were nuclear and two were lysosomal (Fig. 3h). This shows that although mitochondrial features are clearly critical to the model’s prediction, nuclear and lysosomal features are still important, with each featuring in the top ten features for all disease subtypes. Finally, we selected the two most important features to the model’s overall prediction, two mitochondrial textural features (TMRM Signal Enhancement Ratio (SER) Valley and TMRM SER Dark), to assess whether there is a notable difference across the five classes. The scatter graphs demonstrate that there is a significant difference in these features across the five classes (Fig. 3j,k; *P* < 0.0001 between all groups), further demonstrating the importance of mitochondrial features in differentiating between the disease states and healthy control.

### Organelle contacts predict broad disease subtypes

**Fig. 4:**
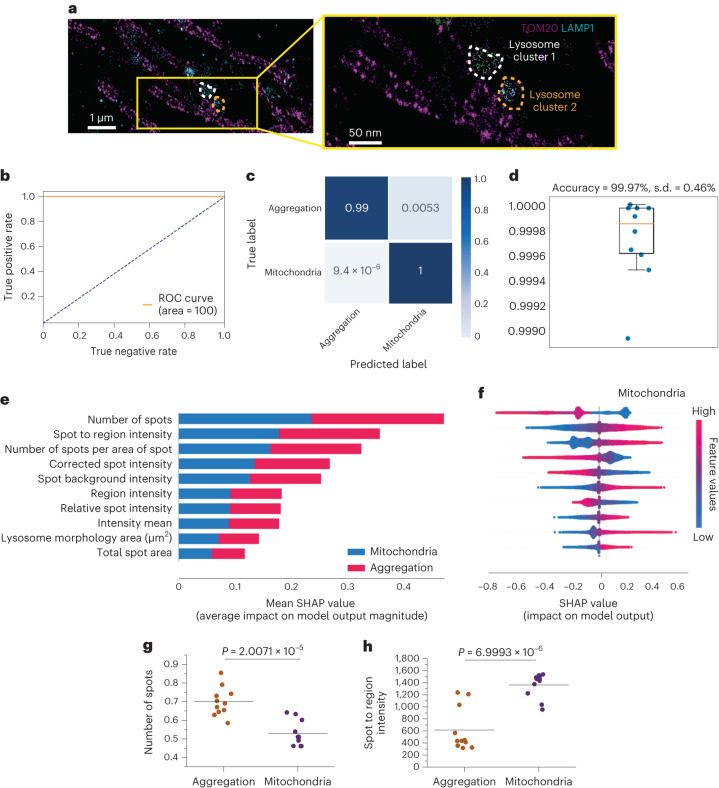
Interaction between cellular organelle networks classifies aggregation and mitochondrial toxicity phenotypes. **a**, Images of mitochondrial and lysosomal co-localization were obtained using super-resolution direct stochastic optical reconstruction microscopy to visualize the contact between the mitochondria and lysosome—organelles both affected in PD^43^. Mitochondria and lysosomes are labelled with TOM20 and LAMP1, respectively (*n* = 2–3 fields of view, across two independent iPSC lines). **b–d**, Receiver operating characteristic–area under the ROC curve of classification performance (**b**); the confusion matrix (**c**); and stratified K-fold cross-validation of the model to identify aggregation versus the mitochondrial toxicity group (**d**) on an unseen test set, trained on the selected cell profile tabular data (mitochondria and lysosome contact; *n* = 10 folds fit and evaluated). Data are presented as mean values ± s.d. The selected tabular data from mitochondria and lysosome co-localization predict the two disease states of mitochondrial toxicity and aggregation with high accuracy (>99%). **e**, Feature ranking that drives the prediction of aggregation on the basis of their SHAP values, coloured by their importance for each class. **f**, A SHAP summary plot of top ten features to classify the groups into mitochondrial toxicity (the SHAP values of the aggregation group have the opposite colours to the mitochondrial group shown here and are therefore not presented). **g**,**h**, Random selection of eight wells to compare the top two lysosomal features that contact mitochondria showing that there is a statistical significance between mitochondrial toxicity and aggregation groups. The statistical details are presented in Supplementary Table 4. Data are presented as data and the mean. Aggregation, combining subtypes of SNCA ×3 and oligomer; mitochondrial toxicity, combining subtypes of complex 1 and mitophagy. Source data

Contact between mitochondria and lysosomes plays a critical role in organellar homeostasis, through damaged mitochondrial clearance (mitophagy) and lysosomal degradation of mitochondrial-derived vesicles. The presence of organellar contacts between mitochondria and lysosomes can be visualized using super-resolution microscopy (Fig. 4a)^25^. We investigated whether contacts between mitochondria and lysosomes—which may reflect the index of mitophagy—would predict the mechanistic subtype. Of note, the use of a membrane potential dye such as TMRM will preferentially detect healthier polarized mitochondria (and not detect damaged depolarized mitochondria).

We trained a machine learning model using overlapping mitochondrial and lysosomal features (that is, mitochondria–lysosome contact areas) and tested whether the model can distinguish the two primary pathologies of aggregation and mitochondrial dysfunction. We combined the SNCA ×3 mutation and oligomer treatment into one single aggregation class; and combined the mitochondrial complex 1 dysfunction and mitophagy into one single mitochondria class (aggregation versus mitochondrial toxicity; the cell profile features and the average scaling factors are shown in Extended Data Figs. 4a and 4b, respectively). This model predicted the correct mitochondrial and aggregation labels at close to 100% and 99% accuracy, respectively, highlighting that the organellar interactions are highly informative at differentiating mitochondrial from aggregation toxicity in PD (Fig. 4b,c and Extended Data Fig. 4c). Stratified K-fold cross-validation exhibited an accuracy of 99.97 ± 0.46% across ten independent runs (Fig. 4d). This model displayed much lower performance at classifying the four subtypes of the two main pathways (Extended Data Fig. 4d). SHAP analysis showed that the number of lysosomal spots and the lysosomal spot to region intensity within mitochondria drive the prediction between aggregation and mitochondrial toxicity (Fig. 4e,f). The top two features identified across plates show a significant change between those two forms of PD (Fig. 4g,h). Thus, a model based on the interaction between two organelles may be a predictor of the broad disease type, distinguishing aggregation from mitochondrial pathologies in PD.

### Convolutional neural network predicts four disease subtypes

**Fig. 5:**
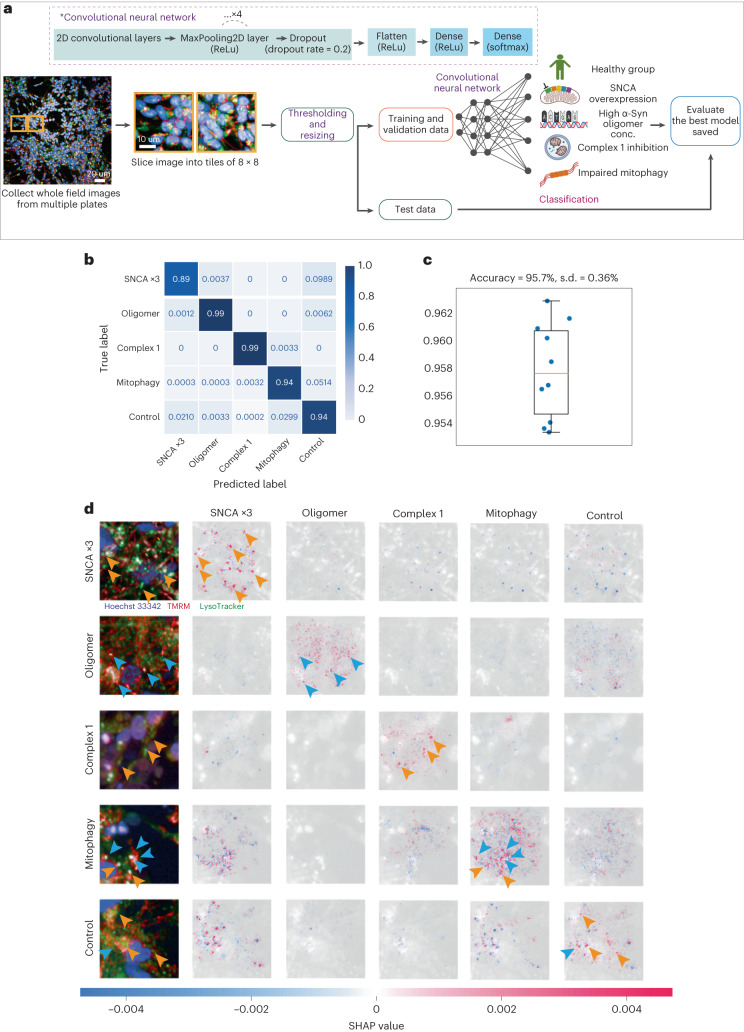
A classifier trained by images using deep neural network accurately discriminates PD pathology. **a**, Illustration of workflow for deep learning with images. **b**,**c**, Deep learning classification performance on an unseen test set trained on 8 × 8 tiled images by the confusion matrix (**b**) and the stratified K-fold cross-validation (**c**) (*n* = 10 folds fit and evaluated). Data are presented as mean values ± s.d. A sample is assigned to five classes with the maximum prediction accuracy (95%). **d**, A SHAP DeepExplainer plot summary. Rows show images from the test set—one from each class—and the columns represent each class. The SHAP value for each score is shown below. Orange and blue arrows indicate either LysoTracker (lysosome) or TMRM (mitochondria) positive areas, respectively.

**Extended Data Fig. 5:**
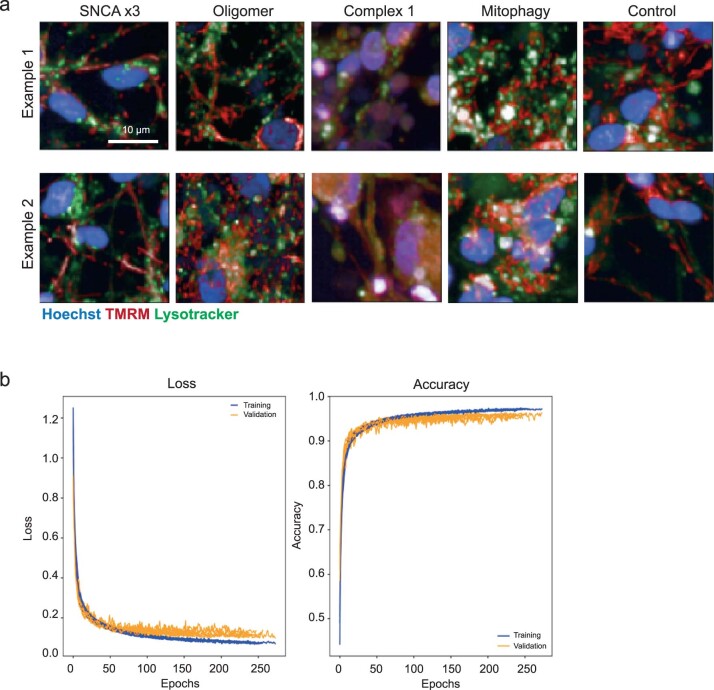
Supportive data for the main Fig. 5. **a**. Examples of 8x8 tiled images with merge of Hoechst, TMRM and Lysotracker images from the test set that are predicted above 99.99% accuracy from each class. SNCA x3 (n = 7983), Oligomer (n = 8307), Complex 1 (n = 11875), Mitophagy (n = 13692), and one control group (n = 22,461) that contained 1–20 cells per sliced image. **b**. The Loss and Accuracy curve (training and validation).

We then created an image-based classifier based on five classes: four disease types and one control. The images were sliced into 8 × 8 tiled images, which contained 1–20 cells per image, allowing preservation of the information contained in neuronal projections and the cell–cell contacts immediately surrounding the neurons (see ref. ^26^ for a review) (see Extended Data Fig. 5a for representative images). We trained a convolutional neural network to distinguish the five classes (Fig. 5a). The confusion matrix from this model shows a true positive rate of >90% for all five classes (average accuracy = 95%, SNCA ×3 = 89%, oligomer = 99%, complex 1 = 99%, mitophagy = 94%, control = 94%; Fig. 5b), highlighting a high accuracy to classify the disease states and control, especially when compared with the previous model trained on cell profiling tabular data (average accuracy = 82%). Stratified K-fold cross-validation showed 95.7% accuracy (Fig. 5c and Extended Data Fig. 5b).

**Fig. 6:**
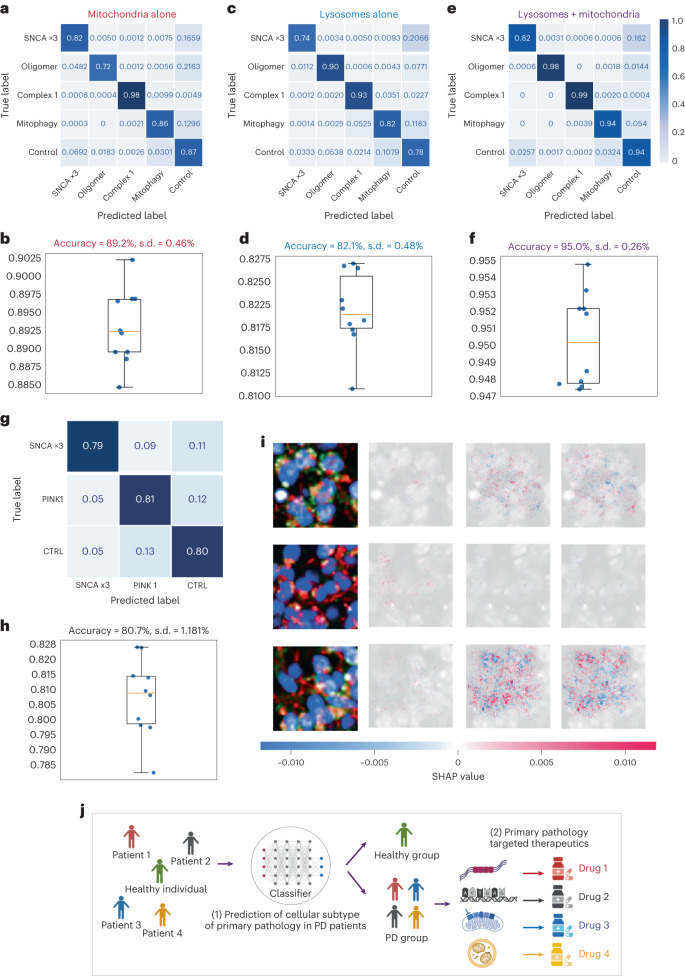
Deep neural network using mitochondria images alone retains high prediction accuracy. **a**–**f**, Deep learning classification performance: confusion matrices and stratified K-fold cross-validation for mitochondria alone (*n* = 10 folds fit and evaluated; data are presented as mean values ± s.d.) (**a**,**b**), lysosomes alone (**c**,**d**), and both together (**e**,**f**). **g–i**, The confusion matrix (**g**) and the stratified K-fold cross-validation (**h**) of the three-class genetic classifier (SNCA ×3, PINK1 and CTRL) on an unseen test set (*n* = 10 folds fit and evaluated; data are presented as mean values ± s.d.). A test sample is assigned to three classes with an overall prediction accuracy of 80.7%, and a SHAP DeepExplainer plot summary is shown (**i**). Rows of the SHAP DeepExplainer plot summary show images from the test set, one from each class, and the columns represent each class. The SHAP value for each score is shown below. **j**, A schematic illustration demonstrates how machine learning-based classifiers can be applied to improve the approach to PD therapeutics. **f(1)**. Our classifier can classify individuals into PD and healthy groups. The PD-diagnosed individuals can be further classified based on their mechanistic subtype. **f(2)**. Mechanism-specific targeting drugs could be matched with PD patients based on their own disease subtypes.

**Extended Data Fig. 6:**
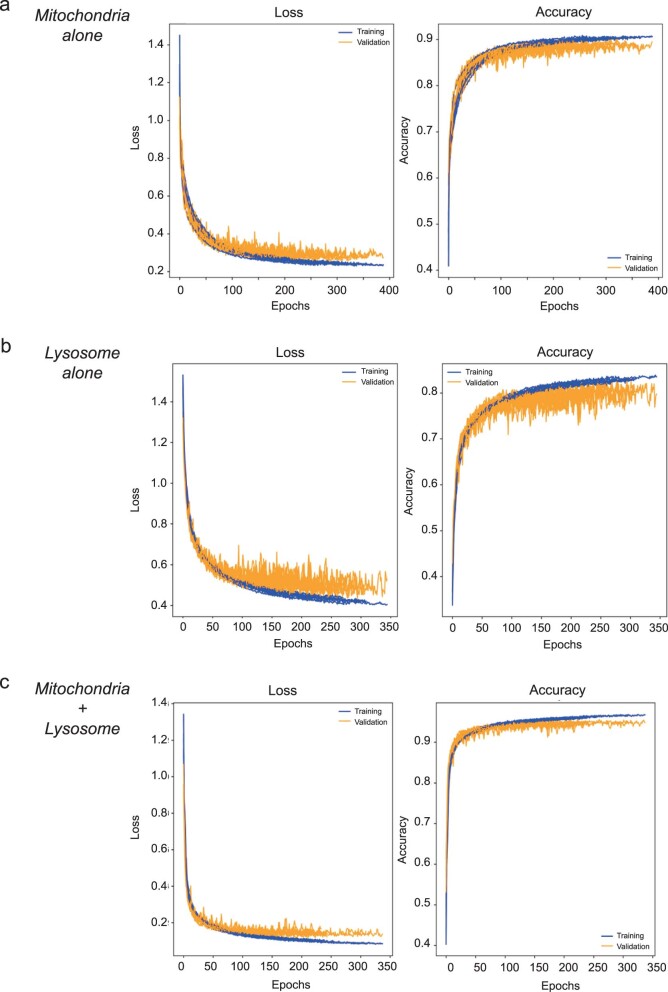
Supportive data for the main Fig. 6. **a–c**. The loss and accuracy curve (training and validation) of the tile-based images of mitochondria alone **(a)**, lysosome alone **(b)** and both together **(c)**.

We next employed SHAP DeepExplainer—a method that indicates how much each pixel contributes to the probability positively or negatively—to understand which aspects of the image are used to generate predictions^24^. Mitochondria, along with the lysosomes, are the major contributors driving the accuracy of the prediction (Fig. 5d). Given the consistent result of the essential role of mitochondria in classifying the disease states, we explored whether images of organelles alone could accurately predict disease states. We trained using images of mitochondria or lysosomes alone or in combination. The true positive rates of the models created for the mitochondria alone, the lysosomes alone and the duet images are 89.2%, 82.1% and 95.0%, respectively (Fig. 6a,c,e and Extended Data Fig. 6a–c); these were cross-validated using stratified K-fold cross-validation (Fig. 6b,d,f). The deep neural network using image data provides a much more robust way to differentiate subtypes of PD and identify which organelle is driving the pathology.

### The effect of a mutation on disease subtype

**Extended Data Fig. 7:**
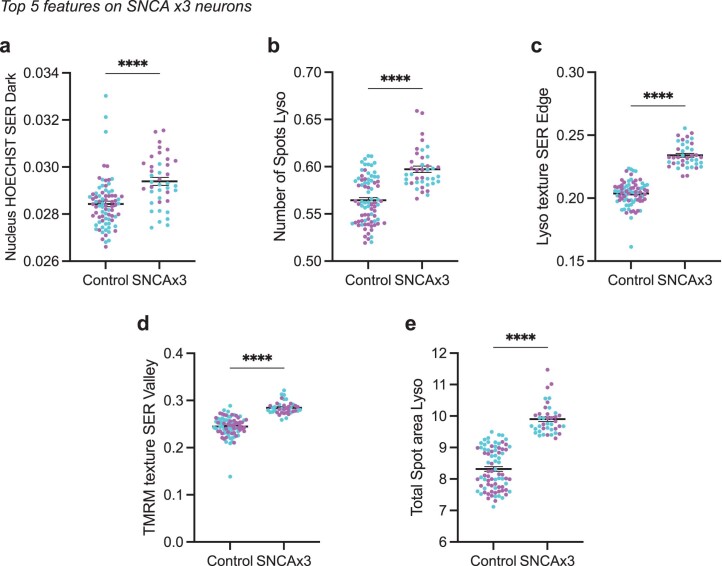
Five top features of newly added line of SNCA. **a–e**, Bar graphs showing the top 5 organellar disease features (Nucleus HOECHSST SER Dark, a; Number of Spots Lyso, b; Lyso texture SER edge, e; TMRM texture SER Valley, d; Total Spot area Lyso, e) for subtype 1 (SNCA x3) (mainly lysosomal) using a different SNCA x3 hiPSC clone and controls. The data points are colour-coded to show independent plates, across control and SNCA x3 neurons (n = 40 wells per genotype across 2 independent plates. The statistical details are found in Supplementary Table 4. Data are presented as data and the mean. Source data

**Extended Data Fig. 8:**
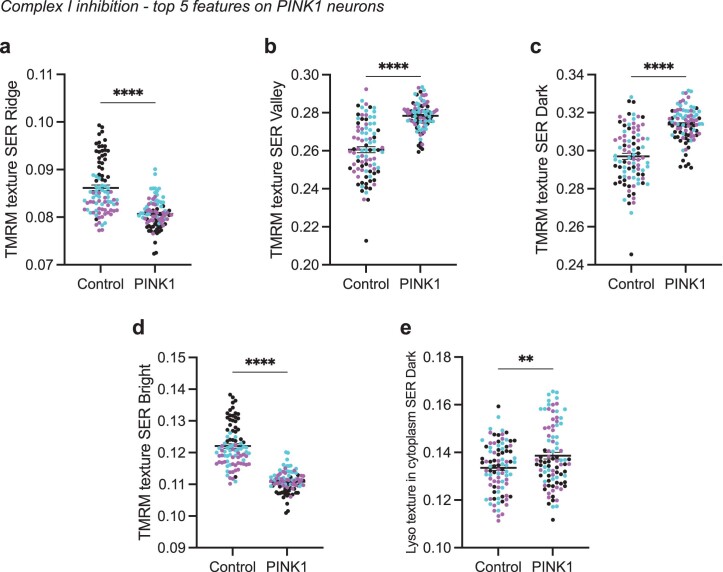
Five top features of newly added line of PINK1. **a–e**, Bar graphs showing the top 5 organellar disease features (TMRM texture SER Ridge, a; TMRM texture SER Valley, b; TMRM texture SER Dark, c; TMRM texture SER Bright, d; Lyso texture in cytoplasm SER Dark, e) for subtype 3 (Complex 1) (mainly mitochondrial) using a hiPSC line from a patient with a PINK1 mutation (ILE368ASN). The data points are color-coded to show independent plates, across control and PINK1 PD neurons (n = 90 wells per genotype across 3 independent plates. The statistical details are found in Supplementary Table 4. Data are presented as data and the mean. Source data

We previously generated disease subtypes mainly on the basis of a genetic mutation or the effect of a chemical compound. Next, we generated a classifier to predict individuals with two different mutations: one that mapped to the protein aggregation pathway (SNCA ×3) and another that mapped to the mitochondrial pathway (PINK1 I368N). The PINK1 mutation has been associated with a range of mitochondrial phenotypes in cellular imaging, including impaired bioenergetics, impaired mitochondrial calcium and impaired mitochondrial clearance^18^ (reviewed in ref. ^27^). First, we extracted the top five features (ranked by the SHAP values) for the previous SNCA ×3 model, and the top five features for the previous complex 1 inhibition model, and tested those features in a new SNCA ×3 line (the same donor, independently derived) (Extended Data Fig. 7a–e) and in a new PINK1 line (Extended Data Fig. 8a–e). The top five features in both disease subtypes exhibited significant differences in the tabular data.

There is no chemical subtype that can accurately mimic a genetic mutation, and so we generated a new image-based genetic classifier to distinguish between the control, SNCA ×3 mutant (proteinopathy) and PINK1 mutant (mitochondriopathy) pathways. Here we incorporated isogenic controls in addition to a healthy control to ensure that the sole effect of the mutation can be predicted, without the confounding effects of different biological/genomic backgrounds. Images were sliced into 8 × 8 tiled images, and we trained a convolutional neural network to distinguish between the three classes. The confusion matrix from this model shows a true positive rate of 81% across all three classes (SNCA ×3 = 79%, PINK1 = 81%, control = 80%; Fig. 6g), demonstrating reasonable accuracy to classify disease mutation and control. Stratified K-fold cross-validation showed an 80.7% accuracy (Fig. 6h). This reduction in performance compared with the chemically induced subtype classifier may be in part due to use of a much smaller dataset (14,531 images), and the use of the PINK1 mutation line under basal conditions (rather than using chemicals to disrupt the mitochondrial pathway). SHAP DeepExplainer demonstrated that, in line with previous classifiers, both the mitochondria and lysosomes contribute to the prediction of the model (Fig. 6e).

## Discussion

Genome-wide association studies have identified multiple genetic risk loci relating to protein homeostasis, protein trafficking, lysosomal function and mitochondrial function in sporadic disease, implicating these pathways in disease pathogenesis^28–30^. Here we used hiPSC-derived cortical neurons—a vulnerable cell type in PD—that robustly recapitulate critical cellular phenotypes of PD to model and define four mechanistic subtypes of disease on the basis of the presence of a familial mutation, proteotoxic stress, mitochondrial stress and induced mitochondrial clearance. Using live high-content imaging, we tracked these disease mechanisms through three key organelles. Our approach is well placed as a preclinical platform to have high predictive value for disease as it is a human model of brain disease in a dish that captures live information on the two critical organelles implicated in PD. This convergence of benefits in our approach enabled us to then develop a highly accurate deep learning classifier that was able to distinguish the presence or absence of disease, and if diseased, the subtype of the disease (see the schematic illustration in Fig. 6f).

Using a range of intensity, morphological and textural features of the mitochondria, lysosomes and nuclei, processed and extracted by the live high-content imaging platform, we trained a model to identify the primary pathology in hiPSC-derived cortical neuronal cultures. We demonstrate that this quantitative feature-based approach is able to predict between control cells and the four disease pathways. Importantly, the use of tabular data in this study enables the ranking of organellar features on the basis of their contribution to the model’s prediction (using SHAP), providing unique and critical insight into the importance of mitochondrial and lysosomal biology in disease. This explainability demonstrates that mitochondrial features contribute most prominently to the overall prediction of the classifier, and specifically the prediction of mitochondrial pathways (complex 1 and induction of mitophagy), with lysosomal and nuclear features also important in the prediction of aggregation pathways (SNCA ×3 and oligomer). Furthermore, when using a similar approach for the interaction between mitochondria and lysosomes, we demonstrate that tabular data based solely on the contacts between these organelles are sufficient to distinguish the two key categories (aggregation and mitochondrial toxicity pathways) with high accuracy. Taken together, these classifiers demonstrate that the information independently contained within the mitochondria and lysosomes and the information contained within their interactions (for example, when lysosomes clear mitochondria) are both sufficient to predict—and are therefore likely to be biologically relevant in—the four mechanisms of disease described here.

Although models trained on tabular data are advantageous due to the level of explainability they provide, they may be susceptible to minor alterations in experimental conditions, such as the loading of imaging dyes and the pre-processing of image data. They may also be dependent on the software processing that converts the images into tabular data which are subject to another filtering level that carries uncertainty with it. As a result, they may lack generalizability. We therefore also generated convolutional neural network (CNN)-based image classifiers using the same large dataset for the tabular data-based model^31,32^. We show that deep CNN-based image classifiers can correctly classify images to accurately identify a disease state from a healthy control state, and this is more generalizable, and shows high performance achieving close to 80–100% accuracy for different disease states.

Our approach offers several advantages to traditional image analysis. Although traditional methods are capable of quantifying well-defined structural properties, and focus on automation and throughput, they do not capture all of the information contained within imaging data. Using traditional image processing software, researchers must typically first choose which feature (or features) to combine to quantify from a vast array of possible cellular phenotypes; this is challenging, time-consuming and may be subject to bias. Machine learning can decipher cellular features at high accuracies in an unbiased manner and achieve greater accuracy than using traditional throughput readouts. Convolutional neural networks have been used successfully via a similar approach to accurately discriminate the progress of neurodegeneration^33,34^.

The high accuracy was achieved through a series of conditions: cell density was relatively high (and necessary) to provide enough information for the training and validation sets, and we also made use of image pre-processing to increase the number of images available to the CNNs. We also trained CNNs with multiple hidden layers to improve the accuracy. Despite the underlying complexity of the deep learning algorithms used, the methods and tools used to classify the images were relatively simple^35^. Only minimal image processing was needed, with each image being divided into 64 individual tiles. Moreover, the use availability and access to a GPU allowed the processing of large amounts of data in a relatively short time (somewhat necessary given the number of images to be processed).

In this study, our approach of creating multiple classifiers based on extracted tabular features and image tiles enables us to gain explainability from the tabular data and translate this to the image classifier by training models on mitochondria and lysosomes individually and in combination. We demonstrate that the loss of nuclear signal (leaving both the mitochondria and lysosomes) in a CNN image-based model does not lower the accuracy of the model. We further demonstrate that a CNN trained on mitochondria alone achieves higher accuracy than the model based on only lysosomes, which is consistent with the feature importance generated from the tabular models.

Our classifiers show high accuracy for chemically induced subtypes of disease, providing proof of concept that our experimental paradigm (namely, integrating patient-derived stem cell neurons with multiplexed organellar imaging data with machine learning approaches) can generate useful predictions about mechanistic subtypes of disease. In the context of the real world, however, patients have different genomic backgrounds with different polygenic risk scores and/or carry rare mutations that may underlie one disease subtype over another. We therefore also generated a classifier based solely on mutations that map to disease subtypes.

In summary, the approach outlined here demonstrates the power of using deep learning in predicting the underlying mechanisms of PD. Importantly, as PD is highly heterogeneous, this platform may enable the disease mechanism in patient cells to be classified. This may have significant clinical implications in both diagnosis and treatment, as the identification of cellular mechanisms may indicate their likely response to proteinopathy (for example, targeting α-Syn) versus mitochondrial (for example, antioxidant therapy) treatments, as illustrated in Fig. 6d. In the future, such a platform may be used to assess which pathway predominates in an individual, and whether specific medications are capable of reversing robust cellular phenotypes, in an unbiased approach^36^.

## Methods

### Generation of human iPSC

We generated neurons derived from hiPSCs that are reprogrammed from two healthy donors (one generated in house, and one commercial line; Thermo Fisher Scientific) or PD patients carrying mutations (SNCA ×3) who had given signed informed consent for the derivation of hiPSC lines from skin biopsy as part of the European Union IMI-funded program Stem-BANCC^37,38^. The isogenic control line of SNCA ×3 was kindly provided from the Kunath laboratory, and was generated using CRISPR/Cas9 editing^37^. PINK1 and the isogenic control lines were purchased from the NINDS Human Genetics Resource Center. The experimental protocol to generate the SNCA ×3 line had approval from the London—Hampstead Research Ethics Committee and R&D approval from University College London, Great Ormond Street Institute of Child Health and the Great Ormond Street Hospital Joint Research Office. The hiPSCs were cultured on Geltrex (Thermo Fisher Scientific) in E8 media (Thermo Fisher Scientific) or mTeSR (Stem Cell Technologies), and passed using 0.5 mM ethylenediaminetetraacetic acid (Thermo Fisher Scientific). All lines were mycoplasma tested (all negative) and short tandem repeat profiled (all matched) performed by the Francis Crick Institute. Neuronal differentiation was performed through dual SMAD inhibition using SB431542 (10 μM, Tocris) and dorsomorphin dihydrochloride (1 μM, Tocris).

### Generation of high-content imaging data and image processing

To generate data live-cell imaging was performed using high-throughput imaging, at day 50 of neural induction, 20,000–40,000 cortical neurons were plated onto a 96-well plate and maintained until use (>day 60). Cells were washed with Hank’s balanced salt solution before loading 10 μM Hoechst (62249, ThermoFisher Scientific), 25 nM TMRM (T668, Thermo Fisher Scientific) and 250 nM LysoTracker Deep Red (L23492, Thermo Fisher Scientific); 500 nM SYTOX green (S7020, Thermo Fisher Scientific) was also added to determine live cells for tabular data profiling. Live-cell images were acquired using an Opera Phenix High-Content Screening System (PerkinElmer). TMRM and LysoTracker labelling were imaged by 516 and 647 nm lasers. SYTOX green labelling was imaged by 488 and 405 nm laser for Hoechst-stained nuclei (17–22 fields of images were taken per well). Live-cell imaging was performed in Hank’s balanced salt solution. Data were collected by Columbus Studio Cell Analysis Software (Columbus 2.9.1, https://biii.eu/columbus-image-datastorage-and-analysis-system).

We collected sets of z-stack images from various focal planes in the *z*-axis while the stage was fixed to the *x*- and *y*-axes. To reduce undesirable effects of out-of-focus features, the training samples consist of two-dimensional information from maximum intensity projections of the z-stack images with fluorescence labels that are pixel registered. The cell profiles based on tabular data and the image dataset were separately collected using the Columbus image storage and analysis system.

All obtained images were transferred to Columbus Image Data Storage and Analysis System (PerkinElmer)—a web interface that provides pipelines to handle high-content screening data. The phenotypic characteristics of each cell are measured, and we selected all available features from the Columbus Image Data Storage and Analysis System. We included the main features for shapes, intensity, texture and microenvironment (for example, relationships between neighbouring cells). Cell features for profiling were extracted by combining modules (for example, ‘find nuclei’, ‘find cytoplasm’, ‘find spots’ for object detection). The defined objects then have a hierarchical structure to detect object features (for example, textures, SER). SER textural features measure patterns in pixel intensity of the ROI (region of interest) (nucleus, mitochondria, lysosome), providing information of fragmentation, networks, and ridges. Together, they provide insights into the structural and pathology of organelles. All lists of the features extracted were described in Extended Data Fig. 3a and the pipelines used are described in Supplementary Table 5. To control cell level quality, we used SYTOX green to define a ‘live cell’, which allows the three organelle features in the same single cell unit by setting a fluorescent threshold; cells showing higher than 500 fluorescent arbitrary units (a.u.) were considered a live cell. The extracted data were exported as csv tables. We acquired the data in two formats: (1) tabular data, which consisted of 56 mitochondrial, lysosomal and nuclear features extracted from the Columbus Image Data Storage and Analysis System (PerkinElmer); and (2) 1,024 × 1,024 raw images, which we further segmented into smaller 8 × 8 tiles.

Organelle features extracted included cell area, expression intensity, the number of spots, roundness, length and width. We also included SER textural features that measure local patterns of pixel intensity providing the structural information of the organelles (see refs. ^39,40^ for reviews).

### Training the classifiers

Tabular data were extracted from multiple plates consisting of four disease groups and a control group using the Columbus Data Analysis and Storage System. For data exploration and feature engineering, five data points, where half the features were missing, were excluded. Of the remaining 177,328 data points, 47 were missing two features, and 1,263 were missing one feature. These missing values were calculated using iterative imputation. This was followed by splitting the dataset into training (*n* = 113,489), validation (*n* = 28,373) and test (*n* = 35,466) datasets. The features (lists are provided in Extended Data 3a) were scaled per control in each plate separately using the Power Transformer scaler. The scaling factor, lambda, was examined in the training dataset, and features with high variance (lambda > 100) were excluded (all features showed lower variances and were therefore all included). We designed and trained a dense neural network with Python using Tensorflow (model structure: three dense layers using ReLU activation, each followed by a dropout layer with dropout rate of 0.2 and a final dense layer with softmax activation). We used adaptive moment estimation as the optimizer, monitoring the validation loss to save the best model and plotting the training and validation losses and accuracy. We used SHAP values to explore the feature importance of our five-class model with a view of gaining insight into the cellular features that drive accurate prediction.

The accuracy of the model was cross-validated using stratified K-fold cross-validation, where the split of the dataset was randomized ten times independently to validate the accuracy of the model. Briefly, data were split into ten stratified folds preserving the percentage of samples of each class across the folds. The model was run ten times, with each fold being used as a test set, and all other folds being used as the training set. Each training set returned by stratified K-fold cross-validation was split further into training and validation datasets using the stratify parameter. The training, validation and test dataset went through the steps of feature scaling followed by creating and training the model as described in the single runs. The validation loss was monitored, and the best model was saved and evaluated on the test dataset. We then average the performance of the ten test datasets and report the mean and standard deviation.

The data to highlight the interactions between mitochondria and lysosomes, was generated with single-molecule localization microscopy (resolution of 20 nm) to visualize the contacts. The tabular data (lists in Extended Data Fig. 4a) generated from five plates (number of cells: controls = 845,143, mitophagy = 644,457, SNCA ×3 = 101,158, α-Syn oligomer = 33,671, complex 1 = 15,751) were processed for data exploration and feature engineering. We then split the data into training (*n* = 1,049,715), validation (*n* = 328,036) and test (*n* = 262,429) datasets. The features were scaled per control in each plate separately using the Power Transformer scaler. The scaling factor, lambda, was examined in the training dataset and one feature (lysosome texture SER Hole 0 px) with high variance across plates (lambda > 50) was excluded from the training, validation and test datasets. The controls were excluded from the datasets after feature scaling resulting in *n* = 508,415 in the training, 159,234 in the validation and 127,388 in the test datasets. We designed and trained a dense neural network using Tensorflow, with the same model structure described for the tabular data above and illustrated in Fig. 3a. We trained with a batch size of 256 and stopped if the validation loss did not improve for 50 consecutive epochs, or if the number of epochs exceeded 500. The same model structure was used to build the classifier to predict the five classes.

For image models, each of the 1,024 × 1,024 pixels high-throughput images, which consisted of 100–400 neuronal cells (each neuron consists of approximately 900–22,500 pixels), was sliced into an 8 × 8 tiled image: SNCA ×3 (*n* = 7,983), oligomer (*n* = 8,307), complex 1 (*n* = 11,875), mitophagy (*n* = 13,692) and one control group (*n* = 22,461) that contained 1–20 cells per sliced image. After cropping, dark images, due to the low numbers of cells contained, were removed by applying a cut-off of 0.1 on the mean intensity threshold and a variance threshold of 0.0275. The image tiles were shuffled and resized to 84 × 84 and split into training (*n* = 51,454) and test (*n* = 12,864) datasets. The training data were then further split into training (*n* = 41,163) and validation (*n* = 10,291) datasets, and shuffled before batching. The neural network was implemented using Tensorflow (see Fig. 5a for the architecture).

### Super-resolution microscopy

To perform single-molecule localization microscopy, neurons were first immunolabelled with mitochondrial and lysosomal antibodies. Neurons were first preserved with 4% paraformaldehyde (PFA) and 0.1% glutaraldehyde for 15 min at room temperature. The neurons were then reduced in 0.1% sodium borohydride in phosphate-buffered saline (PBS) for 7 min, followed by two washes with PBS. They were then permeabilized with 0.5% triton X-100 in PBS for 10 min at room temperature, followed by 1 h of incubation in a blocking solution (3% bovine serum albumin in PBS). After blocking, the neurons were incubated with primary antibodies (TOM20 1:100; Santa-Cruz, sc-17764 and LAMP1 1:100 Cell Signaling Technologies, 9091) made up in blocking solution overnight at 4 °C. After primary antibody incubation, the neurons were washed three times with PBS and incubated with secondary antibodies (Abcam, mouse AF647, Biotium, rabbit CF568; both 1:100 dilution) made up in blocking solution for 1 h at room temperature. The neurons were again washed three times with PBS before post-fixation with 4% PFA for 5 min at room temperature. The samples were then washed twice with PBS before imaging.

The Nanoimager from Oxford Nanoimaging (ONI) was used to perform single molecule localization microscopy. The microscope is equipped with an Olympus 1.4 NA 100× oil immersion super apochromatic objective. Before imaging, colour channel mapping was performed to calibrate the laser channels using 0.1 μm Tetraspeck beads (Thermo Fisher Scientific). The stage was warmed to approximately 25 °C and the illumination angle of the laser was set at 51°. Direct stochastic optical reconstruction microscopy was performed with a photo-switching buffer (B cubed, ONI) to stochastically keep fluorophores in ‘on’ and ‘off’ cycles. An imaging set up of 10,000 frames at 30 ms interval per laser channel with 100% power for the 640 nm and 561 nm lasers was used to super-resolve TOM20 and LAMP1. After imaging, the data was uploaded onto the online visualization and analysis software CODI (ONI, https://pages.oni.bio/codiadvanced-ev-characterisation-made-simple) to produce super-resolved localizations of TOM20 and LAMP1.

### Aggregation of α-Syn

α-Syn monomer was ultracentrifuged (Beckman OptimaMax) at 90,000 g for 60 mins at 4 °C to remove pre-formed aggregates. The protein concentration of the supernatant was determined from the absorbance at 275 nm using an extinction coefficient of 5,600 M^−1^ cm^−1^. The protein was diluted in PBS to a total protein concentration of 70 μM. The aggregation mixture was kept in DNA LoBind microcentrifuge tubes (Eppendorf) and left shaking (200 r.p.m) at 37 °C in an incubator (StuartScientfic) for the duration of the experiment. Aliquots were taken at a series of timepoints over the incubation period and were immediately snap-frozen in liquid nitrogen. Timepoints were stored at –80 °C until required for analysis.

### SAVE imaging

For SAVE imaging, 22 × 40 mm 0.1 mm thickness coverslips (VWR, 6310135) were plasma cleaned (Diener Zepto plasma cleaner) with an argon ion plasma for 1 h to remove fluorescent organic material. The slides were then affixed with 9 × 9 mm well gaskets (Biorad, SLF0201) and 50 μl poly-L-lysine (Sigma-Aldrich, 25988-63-0) was added, incubated for 30 minutes, and subsequently washed three times with 0.02 μm-filtered buffer; 70 μM aliquots were recovered from −80 °C and thawed on ice before being diluted to a concentration of 2 μM into 5 μM thioflavin T in 0.02-μm-filtered 25 mM Tris (pH 7.4) with 100 mM NaCl. Imaging was performed on a custom-built total internal reflection fluorescence microscope described elsewhere^1^. Images were recorded at 50 frames s^−1^ for 100 frames with 405 nm illumination (150–200 W cm^–^^2^).

### Analysis of SAVE images

Data analysis was performed using a custom-written script in Python v.3.8 (code available at: 10.5281/zenodo.7276333). For each image, the stacks were first averaged over 100 frames, and the background was subtracted. Fluorescent species were detected by applying a threshold of five standard deviations above the mean image intensity, and were subsequently analysed using the measuring module (skimage v.0.18.1).

### Immunohistochemistry

Cells were fixed in 4% paraformaldehyde and permeabilized with 0.2% Triton-100; 5% bovine serum albumin was used to block nonspecific binding. Cells were incubated with primary antibodies for 1 h at room temperature and washed three times with 5% bovine serum albumin. Cells were incubated with secondary antibody for 1 h at room temperature. Cells were imaged with PBS after three wash times. Hoechst 33342 (Thermo Fisher Scientific) was added in the second wash if required. Cells were mounted with an antifading medium and left to dry overnight. Images are obtained using confocal microscopy Zeiss LSM 710 (or 880 with an integrated META detection system). The antibodies used are listed in Supplementary Table 6.

### Statistical analysis

Statistical analysis was performed using Origin 2021 (Microcal Software, https://www.originlab.com) software and Prism 8 (https://www.graphpad.com/features). When the decision was made not to reject normality at 5% level, statistical tests were performed using an unpaired, two tailed *t*-test (to compare two individuals) or one-way ANOVA (to compare more than two individuals) corrected with post hoc Tukey. Shapiro–Wilk and Kolmogorov–Smirnov normality tests were used to assess the normality of data. Sample sizes for statistical analysis were selected to capture technical variation including numbers of cell/field of view. Experimental data are represented as mean ± s.e.m. and *P*-value is set at 0.05; *n* = number of wells, if not stated otherwise.

### Reporting summary

Further information on research design is available in the Nature Portfolio Reporting Summary linked to this article.

## Supplementary information

Reporting Summary

Supplementary Tables Supplementary Table 1: information of the hiPSC lines that have been used in this study. Supplementary Table 2: detailed information of the imaging plates used for Figs. 3–5. Supplementary Table 3: detailed information of the imaging plates used for the additional in Fig. 6. Supplementary Table 4: statistical details used in this study. Supplementary Table 5: the analysis was performed with the Harmony software (PerkinElmer) by using a supervised linear classifier. The analysis pipeline and intensity, morphology and texture features used in the classification are listed. Supplementary Table 6: details of the antibodies used for this study.

## Data Availability

Image processing pipelines, all tabular data, whole images (before tiling) and a dataset for the demonstration are publicly available as deposited in Zenodo (10.5281/zenodo.741942)^41^. Source Data are provided with this paper.

## Source data

Source Data Fig. 1 Statistical source data for Fig. 1.
Source Data Fig. 2 Statistical source data for Fig. 2.
Source Data Fig. 3 Statistical source data for Fig. 3.
Source Data Fig. 4 Statistical source data for Fig. 4.
Source Data Extended Data Fig. 7 Statistical source data for Extended Data Fig. 7.
Source Data Extended Data Fig. 8 Statistical source data for Extended Data Fig. 8.

## Code availability

The codes generated for the models and the demonstration are available in GitHub via the Zenodo repository (10.5281/zenodo.8116411)^42^.
