# Supplementary material for: Prediction of mechanistic subtypes of Parkinson’s using patient-derived stem cell models
Source: Nat Mach Intell. 2023 Aug 10;5(8):933–46. doi: 10.1038/s42256-023-00702-9 (PMC10442231; doi:10.1038/s42256-023-00702-9)
Supplement: Supplementary file 1 — Reporting Summary [file 42256_2023_702_MOESM1_ESM.pdf]

nature portfolio

Corresponding author(s): Minee Choi
Last updated by author(s): May 07, 2023

Reporting Summary

Nature Portfolio wishes to improve the reproducibility of the work that we publish. This form provides structure for consistency and transparency in reporting. For further information on Nature Portfolio policies, see our Editorial Policies and the Editorial Policy Checklist.

Statistics

For all statistical analyses, confirm that the following items are present in the figure legend, table legend, main text, or Methods section.

n/a	Confirmed	
☐	☒	The exact sample size (*n*) for each experimental group/condition, given as a discrete number and unit of measurement
☐	☒	A statement on whether measurements were taken from distinct samples or whether the same sample was measured repeatedly
☐	☒	The statistical test(s) used AND whether they are one- or two-sided<br>*Only common tests should be described solely by name; describe more complex techniques in the Methods section.*
☐	☒	A description of all covariates tested
☐	☒	A description of any assumptions or corrections, such as tests of normality and adjustment for multiple comparisons
☐	☒	A full description of the statistical parameters including central tendency (e.g. means) or other basic estimates (e.g. regression coefficient) AND variation (e.g. standard deviation) or associated estimates of uncertainty (e.g. confidence intervals)
☐	☒	For null hypothesis testing, the test statistic (e.g. *F*, *t*, *r*) with confidence intervals, effect sizes, degrees of freedom and *P* value noted<br>*Give P values as exact values whenever suitable.*
☒	☐	For Bayesian analysis, information on the choice of priors and Markov chain Monte Carlo settings
☒	☐	For hierarchical and complex designs, identification of the appropriate level for tests and full reporting of outcomes
☒	☐	Estimates of effect sizes (e.g. Cohen's *d*, Pearson's *r*), indicating how they were calculated

Our web collection on statistics for biologists contains articles on many of the points above.

Software and code

Policy information about availability of computer code

Data collection	Opera Phenix Plus High-Content Screening System, LSM 880 Confocal microscope (Zeiss) and the ZEN software package (Zen black 2.1 SP3, Zeiss)
Data analysis	Columbus™ Image Data Storage and Analysis System, FIJI-win64 (ImageJ 1.53t), Origin 2021, Python 3.9, GraphPad Prism 8, skimage v0.18.1, CODI software. The codes for the models and the Demo are available on Github (https://github.com/Minee-Liane-Choi/chemPredPD). Custom codes used for data analysis is available in https://doi.org/10.5281/zenodo.7276333.

For manuscripts utilizing custom algorithms or software that are central to the research but not yet described in published literature, software must be made available to editors and reviewers. We strongly encourage code deposition in a community repository (e.g. GitHub). See the Nature Portfolio guidelines for submitting code & software for further information.

Data

Policy information about availability of data

All manuscripts must include a data availability statement. This statement should provide the following information, where applicable:

- Accession codes, unique identifiers, or web links for publicly available datasets
- A description of any restrictions on data availability
- For clinical datasets or third party data, please ensure that the statement adheres to our policy

Image processing pipelines, all tabular data, whole images (before tiling) and a data set for the Demo are publicly available as deposited in Zenodo (doi.org/10.5281/zenodo.7419421).

Human research participants

Policy information about studies involving human research participants and Sex and Gender in Research.

Reporting on sex and gender	N/A
Population characteristics	N/A
Recruitment	N/A
Ethics oversight	N/A

Note that full information on the approval of the study protocol must also be provided in the manuscript.

Field-specific reporting

Please select the one below that is the best fit for your research. If you are not sure, read the appropriate sections before making your selection.

☒ Life sciences ☐ Behavioural & social sciences ☐ Ecological, evolutionary & environmental sciences

For a reference copy of the document with all sections, see nature.com/documents/nr-reporting-summary-flat.pdf

Life sciences study design

All studies must disclose on these points even when the disclosure is negative.

Sample size	Sample sizes for experiments were selected to capture (1) technical variation, including numbers of cell/field of view and coverslips and (2) biological variations, including independent inductions and clones or patient line for hiPSC derived neurons.<br>Sample sizes were not predetermined but are similar to those reported in our previous publications (Ludtmann et al., 2018 Nat. Communs. Virdi et al., npj Parkinson's Dis 2022, Angelova et al., Cell death & Differ 2020)
Data exclusions	No data were excluded.
Replication	All experiments were independently repeated 2 – 3 times and all replications are successful.
Randomization	Numbers for cell lines were randomly allocated for each plating. The order of samples to perform experiments was randomized for each experiment to minimize potential effects (e.g. live-cell imaging probe).
Blinding	All experiments were performed as blinding as much as possible. However when blinding is not possible, data were collected and analyzed without bias.

Reporting for specific materials, systems and methods

We require information from authors about some types of materials, experimental systems and methods used in many studies. Here, indicate whether each material, system or method listed is relevant to your study. If you are not sure if a list item applies to your research, read the appropriate section before selecting a response.

Materials & experimental systems

n/a	Involved in the study
☐	☒ Antibodies
☐	☒ Eukaryotic cell lines
☒	☐ Palaeontology and archaeology
☒	☐ Animals and other organisms
☒	☐ Clinical data
☒	☐ Dual use research of concern

Methods

n/a	Involved in the study
☒	☐ ChIP-seq
☒	☐ Flow cytometry
☒	☐ MRI-based neuroimaging

Antibodies

Antibodies used

Anti-MAP2 (abcam, ab183830, 1:500), Anti-TRB1 (abcam, ab31940, 1:500), Anti-CTIP2 (abcam, ab138501, 1:250), TOM20 antibody (Santa Cruz, sc-17764, 1:100), LAMP1 (Cell Signaling Technologies, 9091, 1:100), Recombinant Anti-Alpha-synuclein (phospho S129, abcam, ab51253, 1:100), Goat Anti-Chicken IgY H&L (Alexa Fluor® 488) (abcam, ab150169, 1:500), Goat Anti-Mouse IgG H&L (Alexa Fluor® 555) (abcam, ab150114, 1:500Goat Anti-Rabbit IgG H&L (Alexa Fluor® 647) (abcam, ab150079, 1:500)

Validation

Anti-MAP2: https://www.abcam.com/products/primary-antibodics/map2-antibody-cpr19691-ab183830.htm1, Ani-TRB1: https://www.abcam.com/products/primary-antibodics/tbr1-antibody-ab31940.htm1, Anti-CTIP2: https://www.abcam.com/products/primary-antibodies/ctip2-antibody-25b6-ab18465.htm1, TOM20: https://www.scbt.com/p/tom20-antibody-f-10?rcqucstFrom=scarch, LAMP1: https://www.cc11signa1.com/products/primary-antibodics/1amp1-d2d11-xp-rabbit-mab/9091, Recombinant Anti-Alpha-synuclein (phospho S129): https://www.abcam.com/products/primary-antibodics/a1pha-synuc1cin-phospho-s129-antibody-cp1536y-ab51253.htm1

Eukaryotic cell lines

Policy information about cell lines and Sex and Gender in Research

Cell line source(s)

hiPSC; human fibroblast reprogrammed iPSC; C1 (source: EDi046-A), C3 (Thermo Fisher Scientific, A18945), C17 (EBiSC WTSli017-B), C19 (EBiSC WTSli019-B), SNCAx3 (StemBANCC, SFC831 (STBCi024-C), SNCAx3/the iso (StemBANCC, AST18), PINK1/the iso (NINDS, ND50093 & ND50100)

Authentication

None of the cell lines used were authenticated.

Mycoplasma contamination

All cell lines tested negative for mycoplasma contamination.

Commonly misidentified lines
(See ICLAC register)

None of the cell lines are commonly misidentified lines.
